# Removal of Spinal Calcified Meningiomas With Piezosurgery: Technical Note on a New Application of a Known Device

**DOI:** 10.1227/neuprac.0000000000000063

**Published:** 2023-10-30

**Authors:** Rossella Rispoli, Stefano Pizzolitto, Barbara Cappelletto

**Affiliations:** ‡ Spine and Spinal Cord Surgery Unit, Department of Neurological Sciences, Presidio Ospedaliero Santa Maria della Misericordia, Udine, Italy;; § Pathological Anatomy Unit, Presidio Ospedaliero Santa Maria della Misericordia, Udine, Italy

**Keywords:** Ossified meningioma, Piezosurgery, Thoracic spine, Case report

## Abstract

**BACKGROUND AND IMPORTANCE:**

Ossified spinal meningiomas are a rare form of spinal tumor. The removal in narrow surgical space is challenging because of their hard consistency and strong adhesion to the neural tissue. These meningiomas are often located in the upper thoracic spine, and sometimes, even the identification of the correct intraoperative level is difficult.

**CLINICAL PRESENTATION:**

We describe the clinical findings, surgical strategies, and histological findings of a patient with a thoracic ossified meningioma.

**DISCUSSION:**

We discuss the technical points, safety, and efficacy of the piezosurgery device in reducing the calcified mass.

**CONCLUSION:**

The device has the potential to reduce the operating time and enhance surgical safety when removing ossified meningiomas.

**ABBREVIATION:**
OSM: ossified spinal meningioma.

Meningiomas constitute 25% of primary spinal tumors and are mostly found in the thoracic spine. Purely ossified spinal meningiomas (OSMs) are rare and account for only 1%–5% of all spinal meningiomas.

Surgery is the primary mode of treatment of OSMs. The treatment of choice is gross total resection; however, OSMs may present adhesions to the surrounding nervous tissue, making the surgery challenging.

Historically, in spine surgery, piezosurgery was used to perform the osteotomies. This device has the following 2 advantages: (1) high tissue selectivity that implies no cutting effect on dura and (2) the bone cutting mode is vibrational, without scratching the surrounding tissue.

We present the case of a purely ossified spinal meningioma. We discuss the safety and efficacy of a piezosurgery device for hard tissue removal without producing injuries of the spinal cord.

## CLINICAL PRESENTATION

A 78-year-old female patient presented with progressive weakness and paresthesia in the lower limbs, gait instability, and sphincter disturbances for approximately 2 months. Neurological examination revealed strength deficit with grade 3/5 for all muscle groups of the lower limbs and hypoesthesia with a sensory level at T4. Reflexes were hyperelicitable with a positive Babinski sign. The physical examination revealed a severe proximal kyphoscoliosis.

### Imaging

**FIGURE 1.**
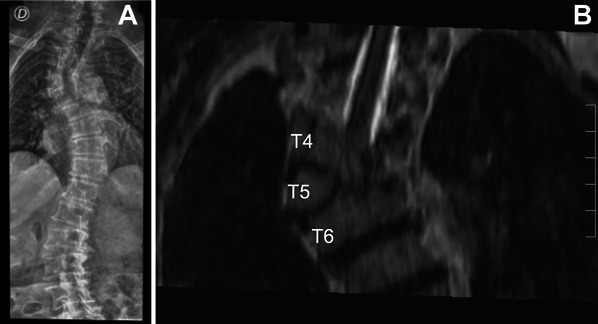
**A**, Antero-posterior thoracic spine x-rays and **B**, coronal T2-weighted MRI showing proximal thoracic right convex scoliosis and right T5 hemivertebra.

**FIGURE 2.**
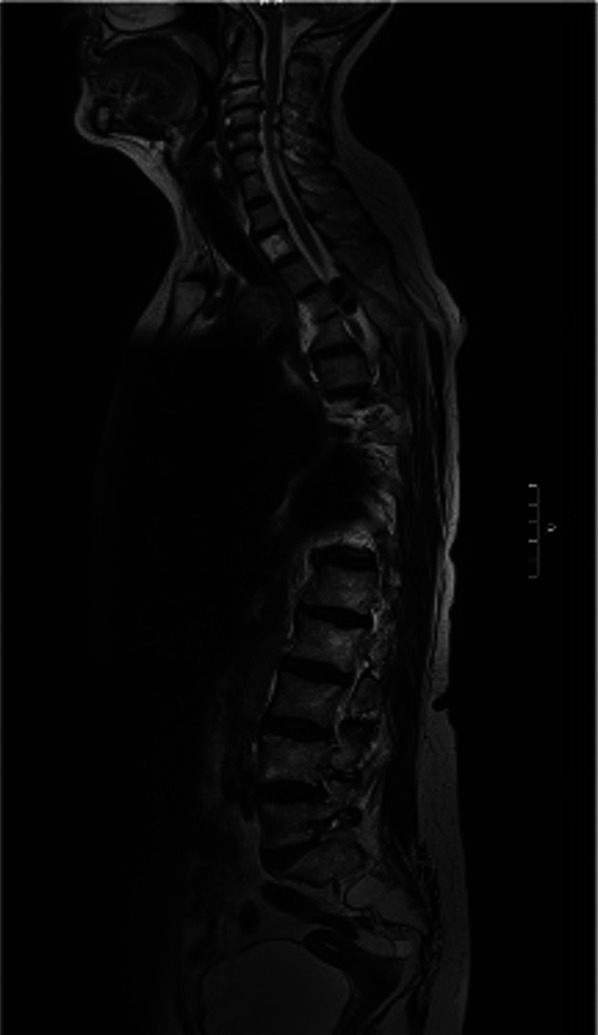
Left side sagittal slice of the whole spine T2-weighted MRI showing the neoplasm at the T4-T6 level in this patient with a T5 right hemivertebra.

**FIGURE 3.**
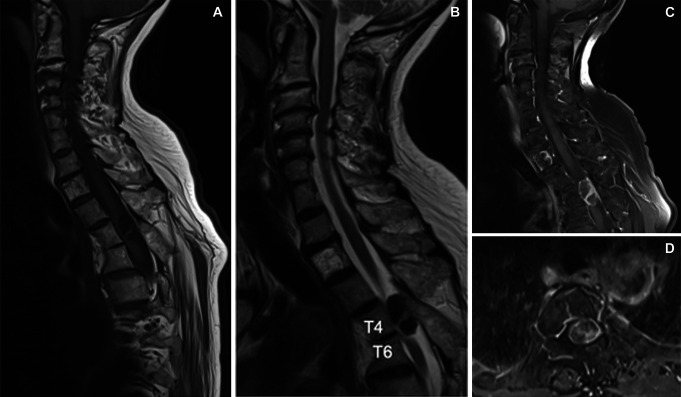
The neoplasm was hypointense on **A**, T1-weighted and **B**, T2-weighted images and peripherally enhanced with gadolinium on **C**, a sagittal and **D**, axial T1-weighted image.

**FIGURE 4.**
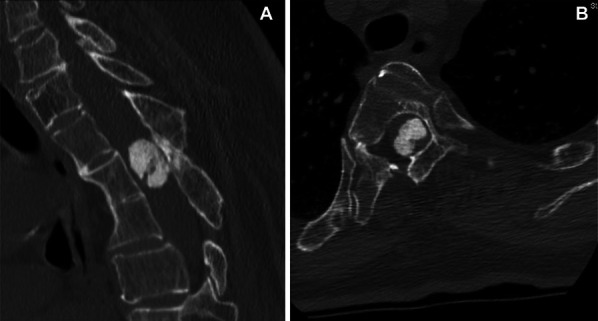
Cervico-thoracic computed tomography scan, using bone algorithm, showing the intradural round calcified mass occupying almost the entire spinal canal on **A**, the sagittal and **B**, axial plane.

In a spoke hospital, a lumbo-sacral spine MRI failed to demonstrate the lesion. A cervico-thoracic MRI showed the lesion, but it was impossible to detect the correct level from both C2 and sacrum. Therefore, a third MRI was performed in our hospital, with sagittal images showing the sacral, lumbar, thoracic, and cervical spine in the same window. The patient showed transition anomaly, proximal thoracic dextro-convex scoliosis, and right T5 hemivertebra (Figure 1). The lesion, consistent with a meningioma, was localized, counting from the cervical spine distally and from the sacrum proximally, at the T4/T6 disk level (Figure 2). The intradural mass was posterior and left sided with respect to the spinal cord, extended between T4 and T6. It showed hypointense signal on T1-weighted and T2-weighted images and peripheral enhancement with gadolinium on T1-weighted images (Figure 3). A cervico-thoracic computed tomography scan showed an intradural round mass, totally replaced by calcification, occupying almost the entire spinal canal (Figure 4).

### Surgery

Under general anesthesia, the patient was positioned in the prone position. We used the Mayfield skull clamp to better secure the skull and have a stable head fixation without external pressure on the eyes and face. Moreover, by positioning the Mayfield head holder, we have the possibility to visualize the spine with the fluoroscopy and obtain ideal intraoperative images to avoid wrong level errors.

Electrophysiological spinal cord monitoring was used for the course of the entire operation.

**FIGURE 5.**
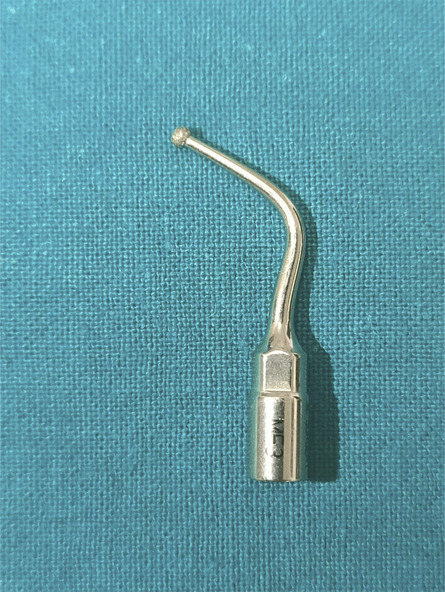
MF3 insert with a diamond-coated ball tip.

We preferred the anteroposterior projection on fluoroscopy as the most effective way of visualizing and counting the vertebrae of the thoracic spine. A midline-skin incision was made followed by 2 levels laminectomy performed with care to preserve the facet joints. Using the operating microscope, a linear midline dura incision was made. The cerebrospinal fluid drainage allowed fine cord retraction. Tumor excision was managed with the Mectron^©^ piezosurgery device (Mectron Medical Technology) that consists of a main unit, which supplies power and has holders for the handpiece and irrigation fluids. We used the MF3 insert with a diamond-coated ball tip (Figure 5), sufficiently angled and gently aggressive to allow the complete demolition of the calcified portion. Once the calcified core of the tumor was demolished, the margins began to fold inward and away from the spinal cord, allowing the complete resection of the remaining mass. Simpson^1^ grade II was achieved. Appropriate hemostasis and watertight dural closure were performed.

The patient consented to the procedure.

### Epilogues

The patient followed a rehabilitative physiotherapy protocol, and her neurological status improved to full motor power in 6 weeks after surgery.

**FIGURE 6.**
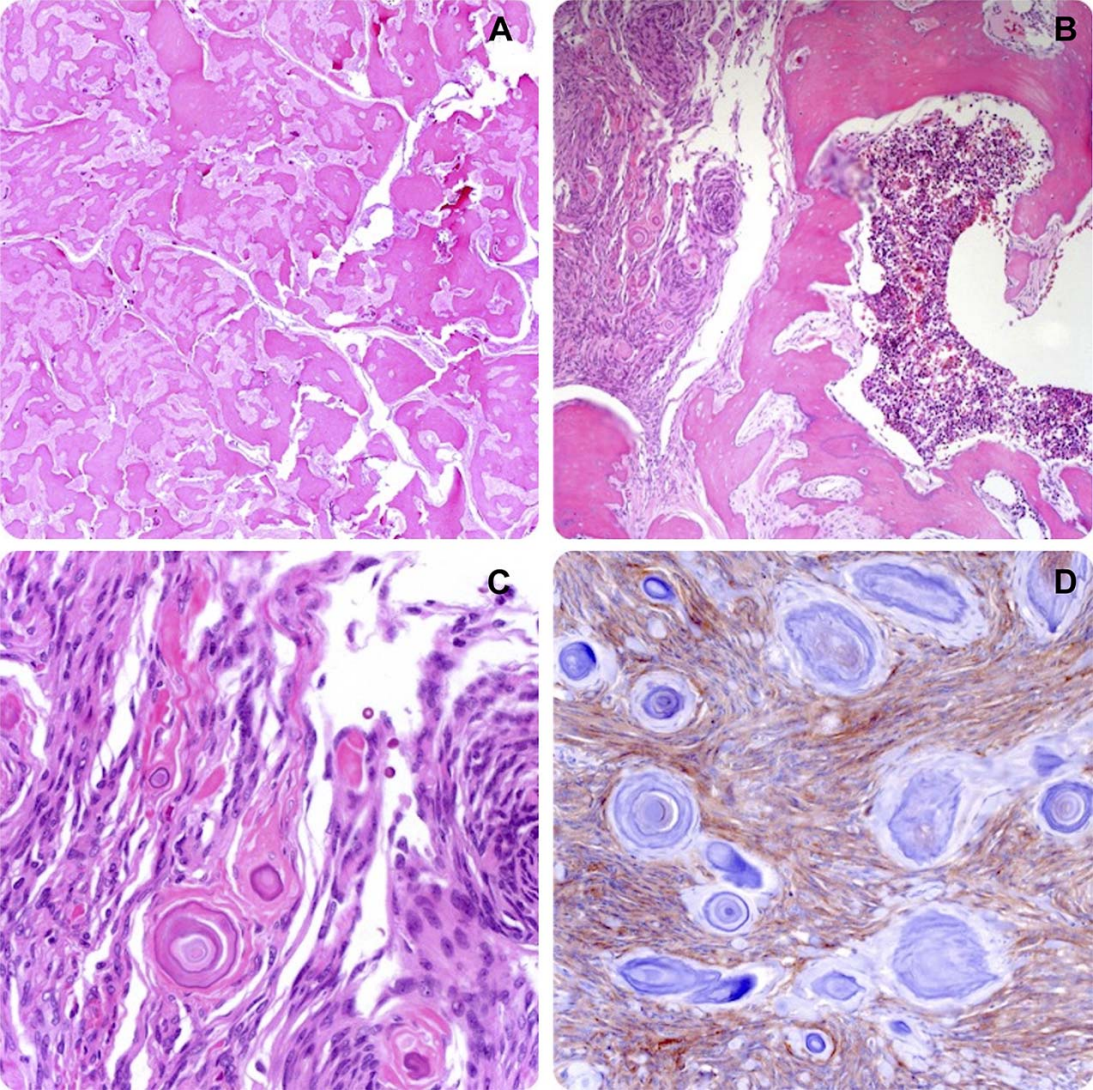
Hematoxylin-eosin staining histopathology showing **A**, a fibroblastic meningioma with extensive mesenchymal osseous component, 100×; **B**, fibrous and psammoma bodies with adjacent trabecular ossification and vascular islands, 100×; **C**, fibrous component with psammoma bodies at higher magnification, 400×; **D**, epithelial membrane antigen immunoreactivity reveals meningothelial cells between psammoma bodies, 400×.

Histopathological examination revealed a metaplastic meningioma with extensive mesenchymal osseous component (Figure 6).

**FIGURE 7.**
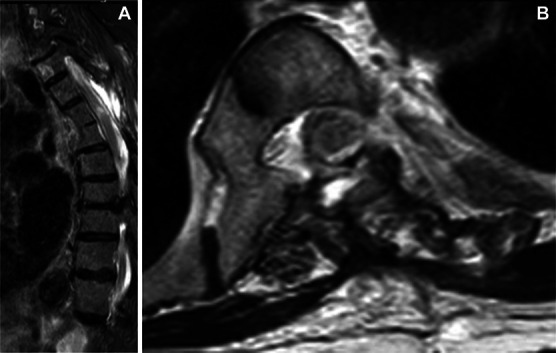
**A**, Sagittal and **B**, axial postoperative T2-weighted MRI showing complete tumor removal.

The 3 months postoperative MRI showed the complete removal of the meningioma (Figure 7).

## DISCUSSION

Ossified meningiomas account for 0.7%–5.5% of all spinal meningiomas.^2,3^ Spinal meningiomas with a hypointense signal on both T1-weighted and T2-weighted MRI images should evoke the possibility of calcified or ossified meningioma. Symptoms are common, and surgical resection is a technically difficult procedure due to the hard consistency of these tumors.^3^ Detection of ossification is important during preoperative planning for safe tumor resection.

The high-signal areas on the computed tomography scan are the radiological features indicative of ossification.^3,4^

The surgical strategy for ossified meningiomas differs from that for other classical cases of meningiomas because central tumor debulking and, if dura needs to be resected, dural reconstruction can be challenging.^3,5,6^ In our case, surgery was performed with the Mectron^©^ piezosurgery device, which we found useful for hard tissue removal, including calcified parts.

Piezoelectric bone surgery, also known as piezosurgery, represents a surgical technique for osteotomy or osteoplasty. The technique underlying the piezoelectric effect and ultrasound technology results from the power of the surgical cutting associated with the possibility to distinguish between hard tissue and soft tissue.^7^ The microvibrations produced by the piezoelectric ceramic cut only mineralized structures, without producing bone frustules and without injuring the soft tissue.^8^

Hidaka first applied piezosurgery osteotomy to spinal surgery in 1998, and since then, it has been found to reduce the incidence of nerve and dura mater injury compared with high-speed drilling both in double-door cervical expansive laminoplasty^9,10^ and in other spinal surgeries. The piezosurgery osteotomy plays a role in bone cutting through mechanical fragmentation effect and cavitation effect, which has the advantages of its good hemostasis, tissue selectivity, and minimal neurovascular damage.^11^

In the literature, there are only 2 cases that report the surgical removal of thoracic calcified meningioma through the utilization of an ultrasonic osteotome (Misonix^©^); the authors claim that this technology has the potential to decrease operative time, operator fatigue, and complications associated with manipulation of the spinal cord.^12^

The main advantage of the Mectron piezosurgery device is that the heat generated by the instrument is greatly reduced compared with other devices. First, the operator can adjust vibration in a scale of 10 possible amplitudes. Second, there is a continuous irrigation of saline that prevents overheating of the surrounding surfaces while maintaining optimal cutting capacity. In the literature, the heat generated by ultrasonic scalpels is comparable with the heat produced by the high-speed drill.^13^ The high temperature could damage the surrounding soft tissues, such as the dura mater or the spinal cord, and some studies reported dural tears with the use of bone ultrasonic scalpels in 1.6%–18% of patients.^14^

Another advantage of the Mectron piezosurgery device is its lightweight handpiece and many different inserts, straight, and angulated. In particular, the angulated diamond ball insert (Figure 5) allowed us to demolish the ossified meningioma under the operating microscope comfortably and precisely handling the device.

We report here, for the first time to our knowledge, the demolition of a spinal calcific meningioma with a piezosurgical device. We have experienced that the Mectron^©^ piezosurgery is a useful instrument, minimizing the risk of mechanical and thermic injury when applied close to neural tissues. The micro ball shaver tip (diameter = 1.5 mm) can be easily introduced in very narrow spaces, causes fragmentation and cavitation in the calcified lesion, and is cooled by continuous irrigation of physiological saline, avoiding excessive heat production. The handpiece is light in weight and can be readily manipulated with 1 hand. Ultimately, this technology has the potential to shorten the operating time and to increase the safety of this delicate surgery.

### Limitations

This study has some limitations. First, the study is based on a single case. Second, the supporting literature is limited due to the rarity of calcific meningiomas. Third, piezosurgery is not widely used in neurosurgery, and the authors were unable to compare a homogenous group treated with different techinques. It is worth nothing that the findings reported in the article offer valuable insights to neurosurgeons dealing this rare condition.

## CONCLUSION

The present case indicates that the Mectron^©^ piezosurgery is a valid tool allowing an effective and secure resection of the spinal calcific meningioma. We recommend the collection of additional experience across a larger group of patients to further assess the device for calcified spinal tumors removal.

## COMMENTS

We read with great interest the technical note brought forth by the authors, who should be commended for describing the resection of an intradural ossified spinal meningioma (OSM) extending between T4-T6 using a piezosurgical device. Under neuromonitoring, a midline incision followed by 2-level laminectomy was performed. The tumor excision was carried out using a piezosurgical device until a Simpson grade II was achieved. The patient completed physical therapy and regained full power at 6 weeks postoperatively. Magnetic resonance imaging at 3 months showed complete tumor resection. While piezosurgical devices have been previously used in spine surgery,^1a^ the authors sought a creative approach by using piezosurgery to excise a rare spinal cord tumor.

Although OSMs present a surgical challenge, approximately 90% of cases can be effectively treated with Simpson's grade-1 resection.^2a^ However, differing opinions exist regarding the optimal approach for safe gross total resection. Some authors recommend using high-speed diamond drills,^3a^ while others have advocated for the use of piezosurgical devices.^[Bibr R4a][Bibr R5a]^ Yamane et al^4a^ reported that a curettage of an OSM with Kerrison rongeurs resulted in temporary motor evoked potentials (MEPs) loss, but switching to an ultrasonic bone aspirator for further tumor resection allowed MEPs to improve while safely taking out the tumor. Conversely, concerns have been raised about using piezosurgery for bony component removal in OSMs, as it may carry a potential risk of neurovascular damage, making it more appropriate for accelerated debulking at the tumor periphery.^[Bibr R2a][Bibr R6a]^ Due to the complexities of central tumor debulking in OSMs,^7a^ it is advisable to first debulk the tumor, and then carefully separate the pia mater and tumor surface before employing the aspirator to avoid neural injury. Furthermore, adjunctive intraoperative neuromonitoring, particularly MEPs and somatosensory evoked potentials (SSEPs), plays a crucial role in detecting and reducing postoperative iatrogenic neurological deficits.^8a^

We extend our gratitude to the authors for their remarkable and insightful study on the application of piezosurgery in OSM removal, shedding light on potential advancements in the field. Investigating larger patient cohorts with long-term follow-up will provide more robust evidence and help establish piezosurgery's role in improving surgical outcomes and reducing complications.

**Hani Chanbour and Scott L. Zuckerman**

*Nashville, Tennessee, USA*
